# Supplementary material for: Cell-to-cell interactions revealed by cryo-tomography of a DPANN co-culture system
Source: Nat Commun. 2024 Aug 16;15:7066. doi: 10.1038/s41467-024-51159-2 (PMC11329633; doi:10.1038/s41467-024-51159-2)
Supplement: Supplementary file 1 — Supplementary Information [file 41467_2024_51159_MOESM1_ESM.pdf]

Supplementary Information for

Cell-to-cell interactions revealed by cryotomography of a DPANN coculture system

Matthew D Johnson[1,2,#], Doulin C Shepherd[1,2,#], Hiroyuki D. Sakai[3], Manasi Mudaliyar[1,2], Arun Prasad Pandurangan[4], Francesca L Short[5], Paul D. Veith[6,], Nichollas E Scott[7], Norio Kurosawa[3], Debnath Ghosal[1,2,*]

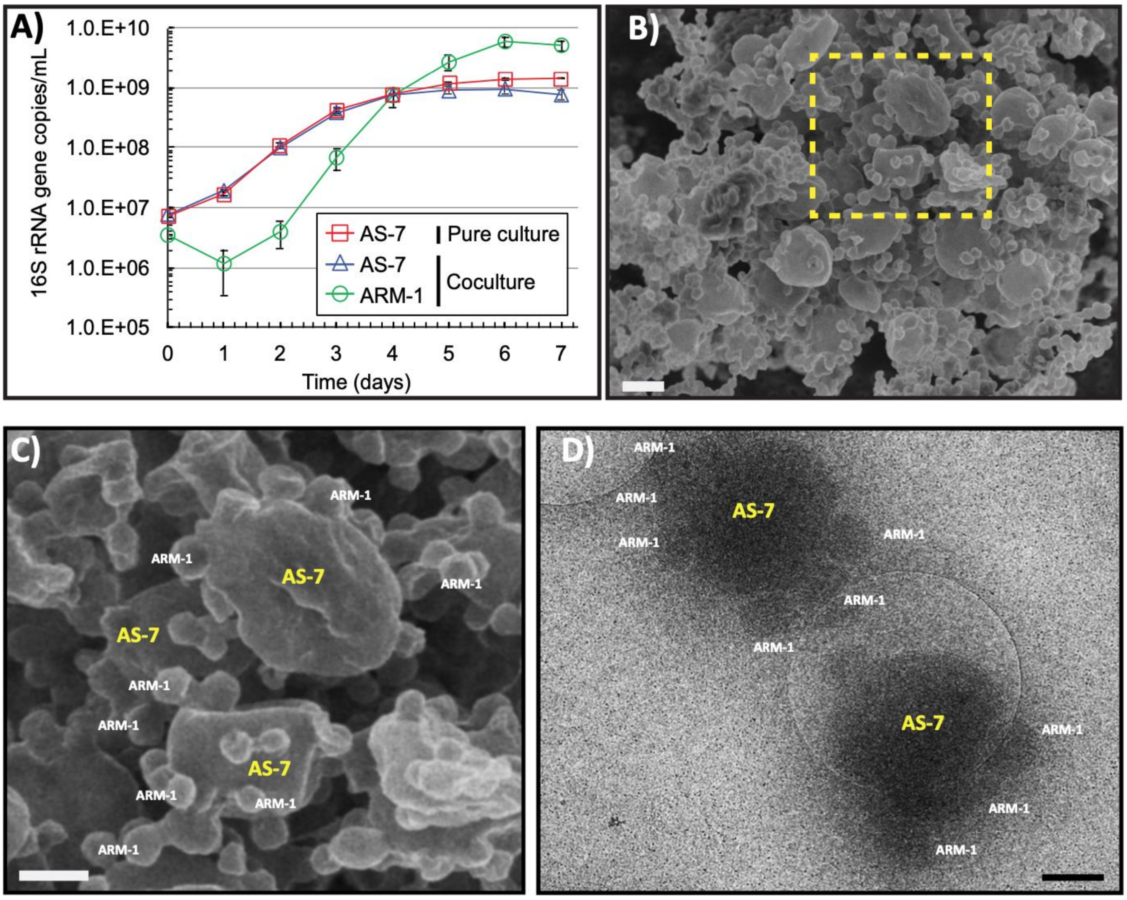

Supplementary Figure 1. The ARM-1 and AS-7 co-culture, growth, and association. (A) Growth curve analysis of pure AS-7 and in co-culture with ARM-1 using 16S-rRNA quantification. (B) SEM analysis of an example co-culture used in this work showing small ARM-1 cells attached to larger AS-7 cells. (C) Zoomed in view of the image shown in (B) (yellow dotted square area) showing multiple ARM-1 cells are attached to each AS-7 cell. (D) Cryo-TEM image of ARM-1 and AS-7 co-culture showing ARM-1 and AS-7 association. Scale bar 500 nm.

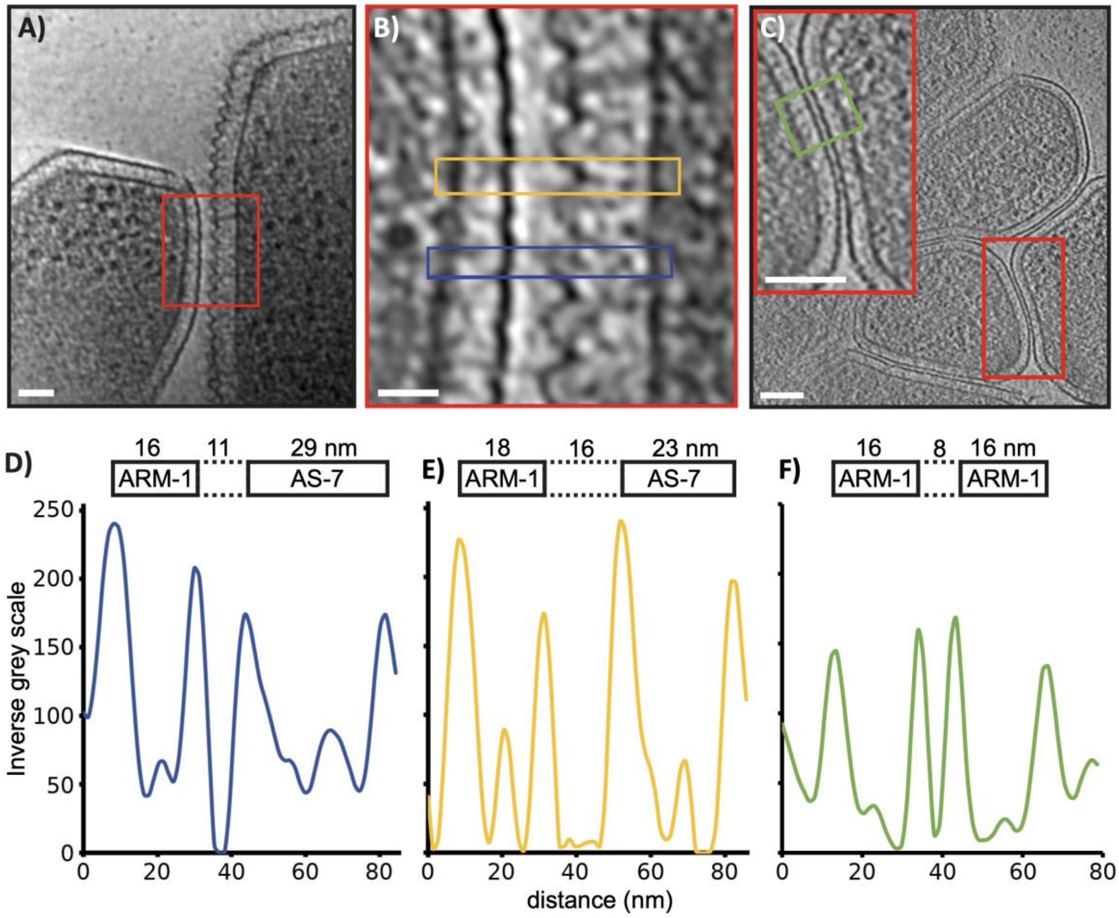

Supplementary Figure 2. **I**nteraction between ARM-1 and AS-7. (A) Tomographic slice of AS-7 and ARM-1 co-culture showing an ARM-1 cell (left) interacting with an AS-7 host cell (right). (B) The red box in (A) is enlarged in (B). High-resolution details of the interface between ARM-1 and AS-7 is visible. The AS-7 surface forms an undulating contour. The distance between ARM-1 and AS-7 varies in peak (blue box) and trough (yellow) regions. (C) Tomographic slice showing interaction between two ARM-1 cells. Inset, zoomed in view of the region within red box showing the distance between two neighbouring ARM-1 cells is consistent along the length of their contact. (D-E) Density profile analyses of tomographic slices showing distance between an ARM-1 and a AS-7 cell at peak (D) and trough (E) regions and between two ARM-1 cells (F). Scale bar 20 nm (A), 20 nm (B), and 50 nm (C) respectively.

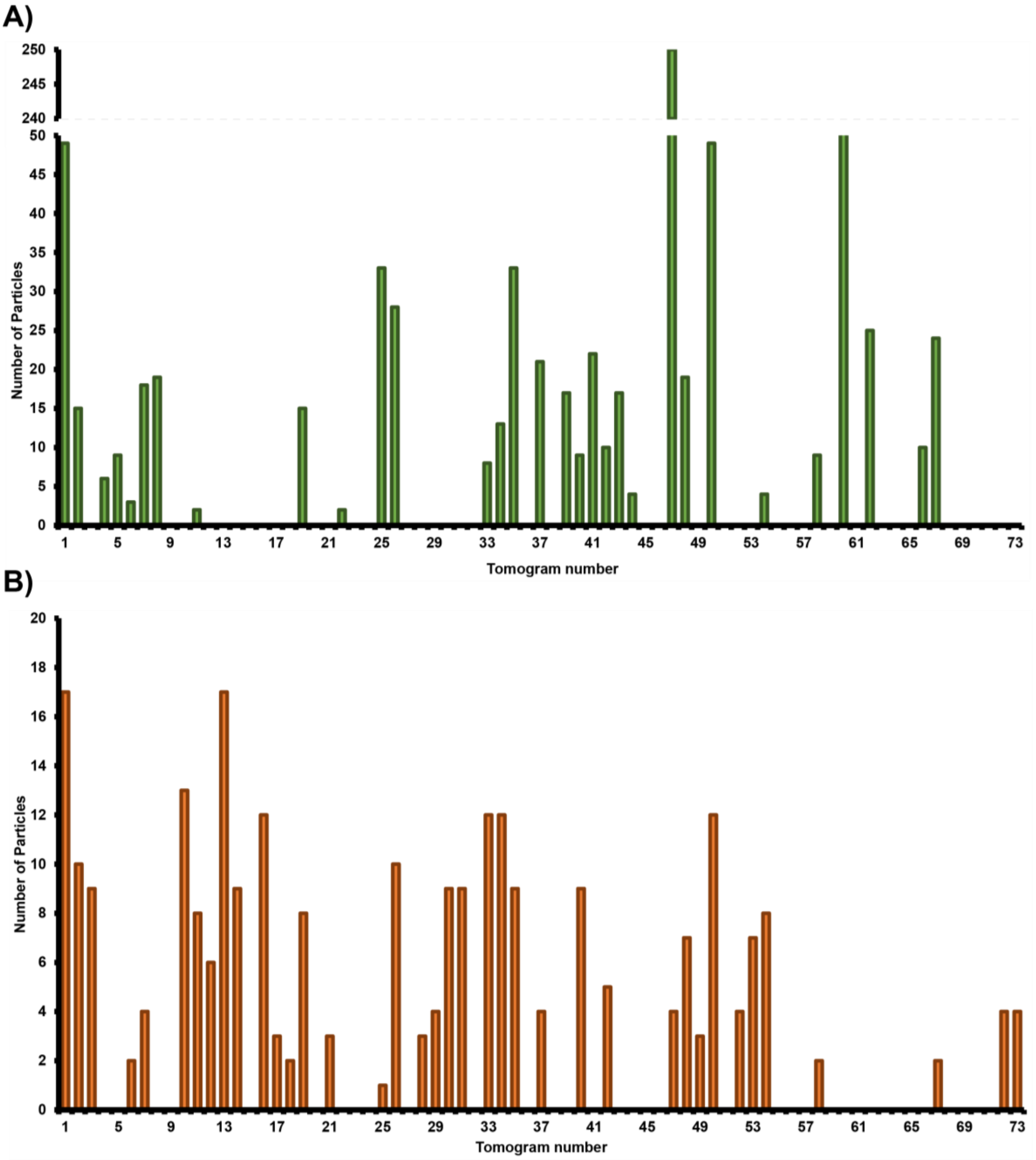

Supplementary Figure 3. Number, frequency and distribution of particles picked from tomograms. **(A)** Number of "top-views" of primed tubes picked from each tomogram. **(B)** Number of extended tubes picked from each tomogram.

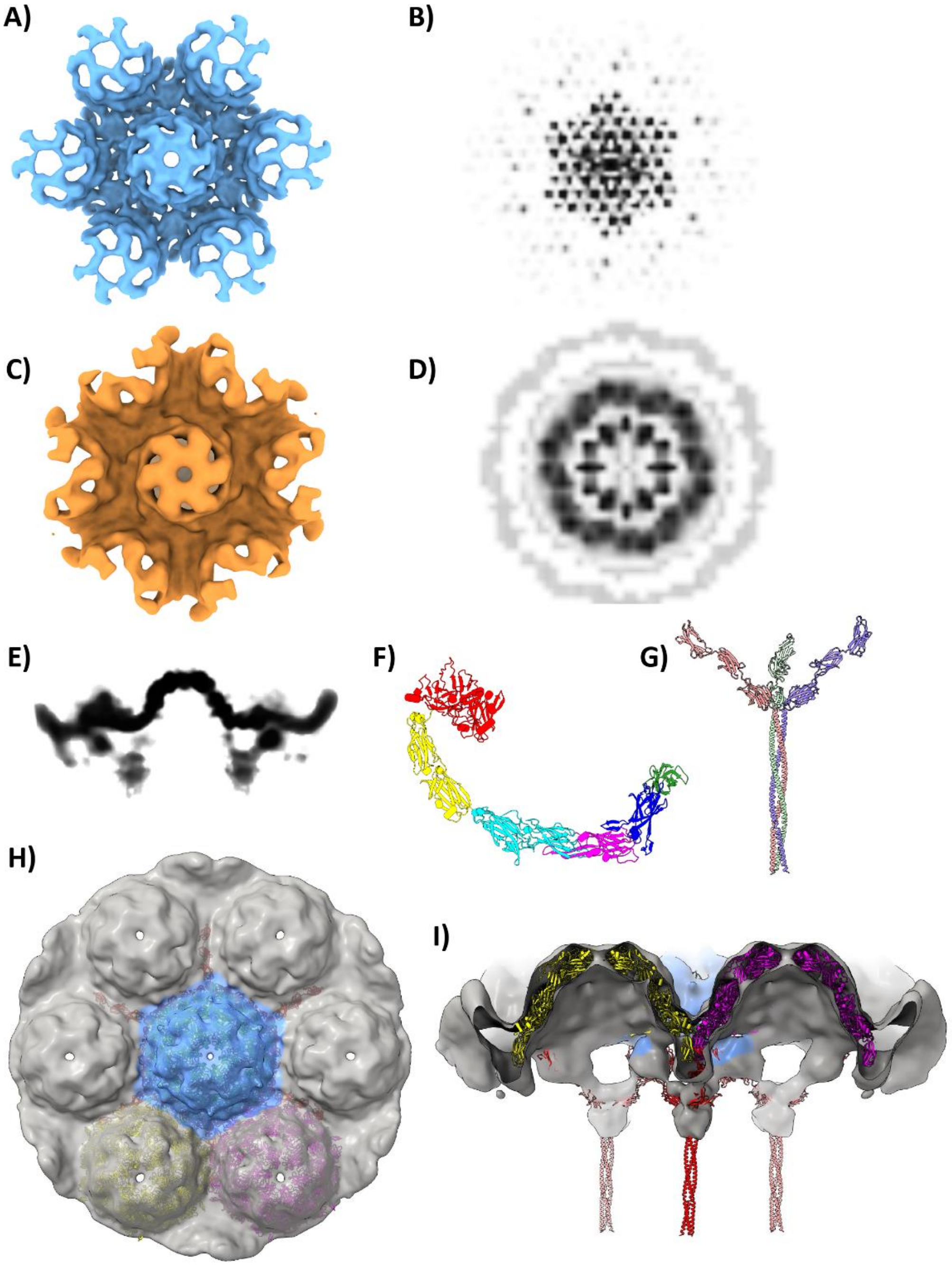

Supplementary Figure 4. In situ structures of the AS-7 S-layer, primed nanotube and their respective power spectrums. (A) Top view of the surface representation of the AS-7 S-layer structure determined by STA. (B) Central cross section of the 3D power spectrum for (A)

revealing clear six-fold symmetry. (C) Top view of the surface representation of the primed AS-7 nanotube. (D) Central cross section of the 3D power spectrum for (C). (E) Orthoplane view of the AS-7 S-layer structure as determined by STA. (F) AlphaFold model of SlaA[1]. Distinct domains marked in different colours - red (28-373), yellow (74-506, 507-639), cyan (640-784, 785-919), magenta (920-1076), blue (1077-1230) and green (1231-1345). Full-length model of SlaA was split into multiple units and fitted into the STA density map in a semi-automated manner [2–4]. (G) AlphaFold multimer model of SlaB trimer[1]. Individual protomer are shown in red, blue and green. (H) Fits of the three representative hexamer models of SlaA shown in blue, yellow and magenta as ribbon representation within the transparent density map[5–7]. (I) Same as (H) rotated 90° about X-axis with cross section showing 4 SlaA proteins in yellow and magenta as well as SlaB trimers in the background.

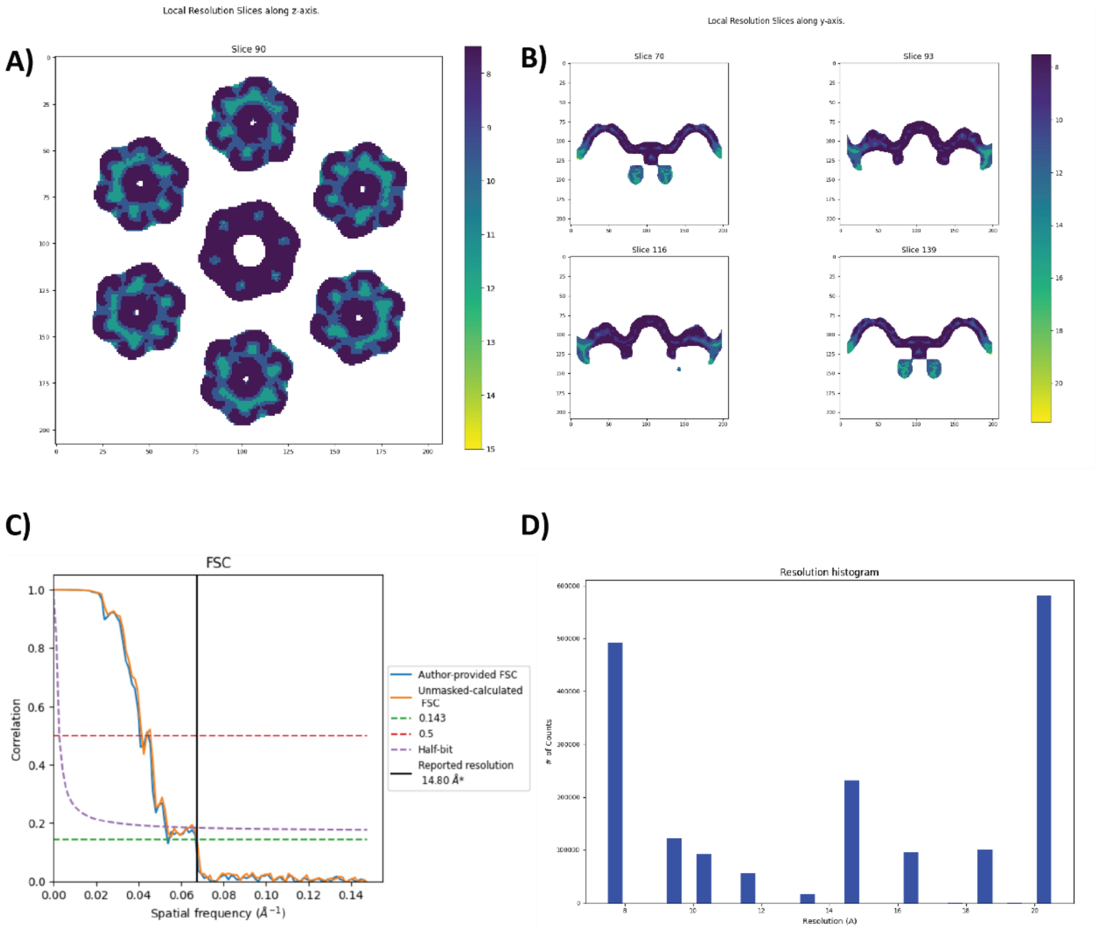

Supplementary Figure 5. Local resolution of the subtomogram averages of AS-7 S-layer calculated by ResMap. (A) ResMap analysis of the AS-7 S-layer structure side cross-sections and top cross-section (B). (C) Fourier Shell Correlation (FSC) curves derived from unmasked half maps of the AS-7 S-layer. The plot compares user provided FSC (Relion4) and the FSC curve generated by the EMDB validation server. (D) Resolution histogram plot generated by ResMap for AS-7 cell S-layer.

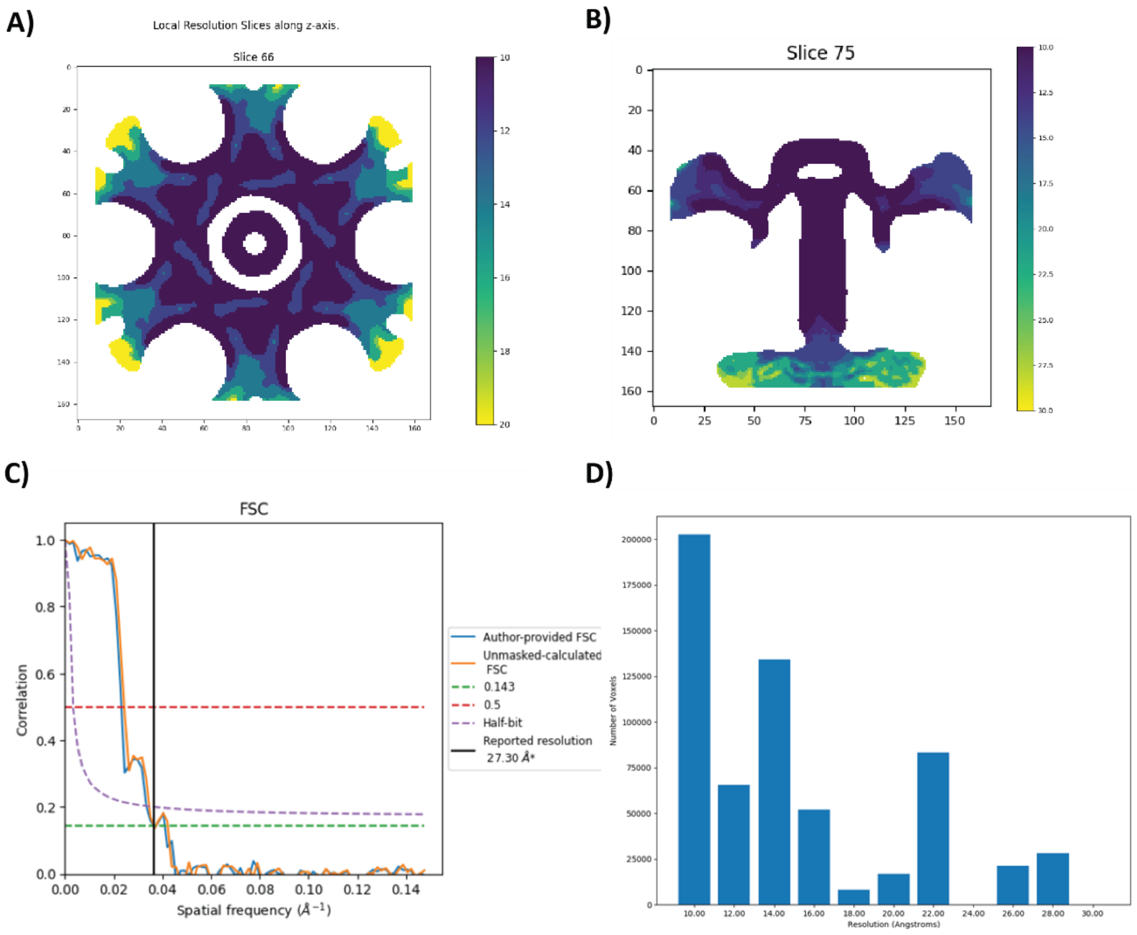

Supplementary Figure 6. Local resolution of the subtomogram averages of AS-7 primed tube calculated by ResMap. (A) ResMap analysis of the AS-7 primed tube structure side cross-sections and top cross-section (B). (C) Fourier Shell Correlation (FSC) curves derived from unmasked half maps of the AS-7 primed tube. The plot compares user provided FSC (Relion4) and the FSC curve generated by the EMDB validation server. (D) Resolution histogram plot generated by ResMap for AS-7 primed tube.

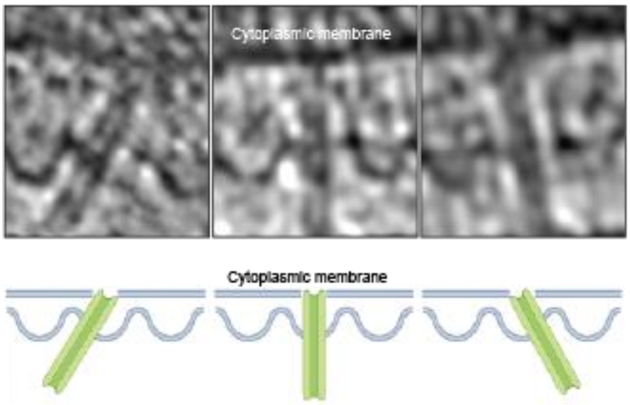

Supplementary Figure 7. Three example particles of extended tube structures demonstrating the variability of their orientation relative to the AS-7 membranes (top). Diagrammatic representations of the tube orientations from the above panels (bottom) created with BioRender.com released under a Creative Commons Attribution-NonCommercial-NoDerivs 4.0 International license.

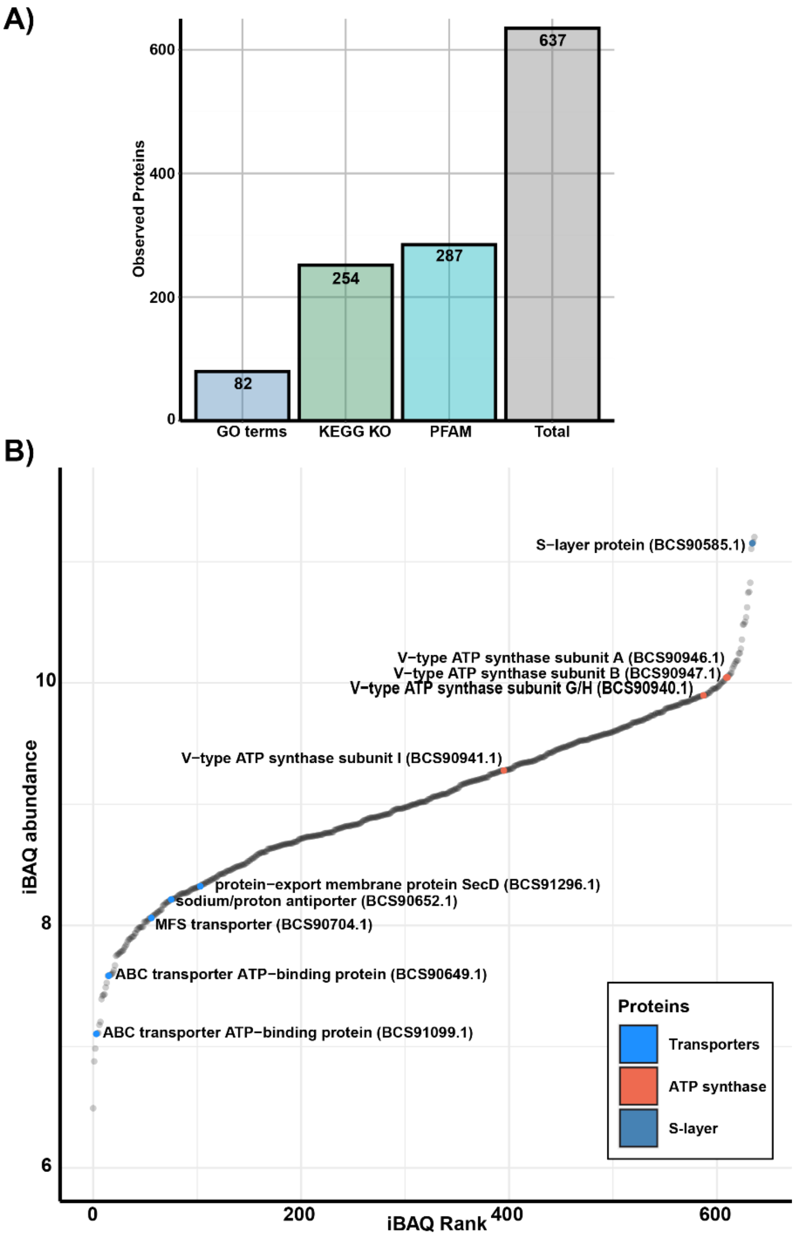

Supplementary Figure 8. Proteomics analysis of ARM-1 during co-culture with AS-7. **(A)** Bar chart of automated annotations of ARM-1 proteins by GO term, KEGG KO, and PFAM. **(B)** ARM-1 protein abundance determined by iBAQ analysis, ATP synthases and transporters are highlighted in red and blue respectively. The ARM-1 S-layer is highlighted in dark blue.

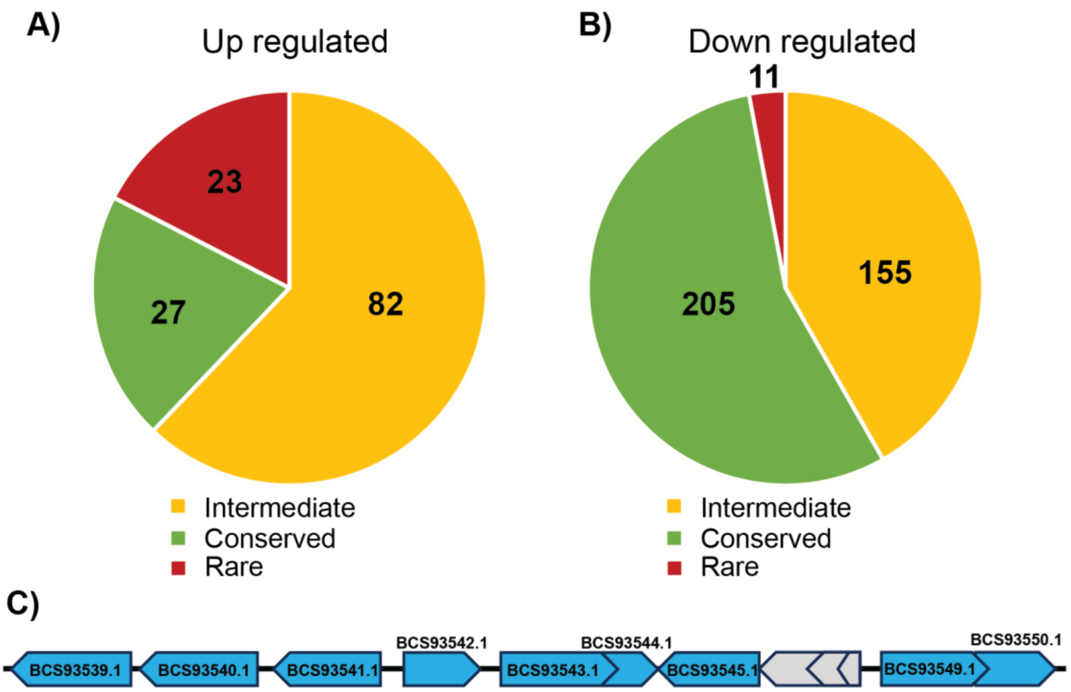

Protein ID	Annotation
**BCS93539.1**	acetolactate synthase
**BCS93540.1**	putative sialic acid transporter
**BCS93541.1**	3,4-dihydroxyphenylacetate 2,3-dioxygenase
**BCS93542.1**	creatinine amidohydrolase
**BCS93543.1**	3-phenylpropionate/cinnamic acid dioxygenase subunit alpha
**BCS93544.1**	hypothetical biphenyl dioxygenase beta subunit
**BCS93545.1**	5-carboxyvanillate decarboxylase
BCS93546.1	*hypothetical protein*
BCS93547.1	*hypothetical protein*
BCS93548.1	*hypothetical protein*
**BCS93549.1**	hypothetical protein
**BCS93550.1**	3-oxoacyl-[acyl-carrier-protein] reductase FabG

Supplementary Figure 9. Comparative genomics analysis of Sulfolobaceae. Genes encoding proteins that were found to differentially regulated by proteomics analysis in this study were compared across the Sulfolobaceae family by presence absence analysis. A complete list of genomes used is in supplementary table 2. Pie charts show the level of conservation of all up regulated (**A**) and down regulated (**B**) proteins. Conserved genes (green) are those found in >80% of Sulfolobaceae genomes, Intermediate genes (yellow) are present in 10-80%, and rare genes

(red) are present in <10% of Sulfolobaceae. (**C**) Gene cluster (ii), which is predicted to be involved in catabolism of aromatic compounds and is found in *M. javensis* but not in other Sulfolobaceae representatives. Genes with upregulated protein products in the presence of ARM-1 are shown in blue, while others are shown in grey. Automated annotations for each protein ID are shown below; these were generated by Prokka[8].

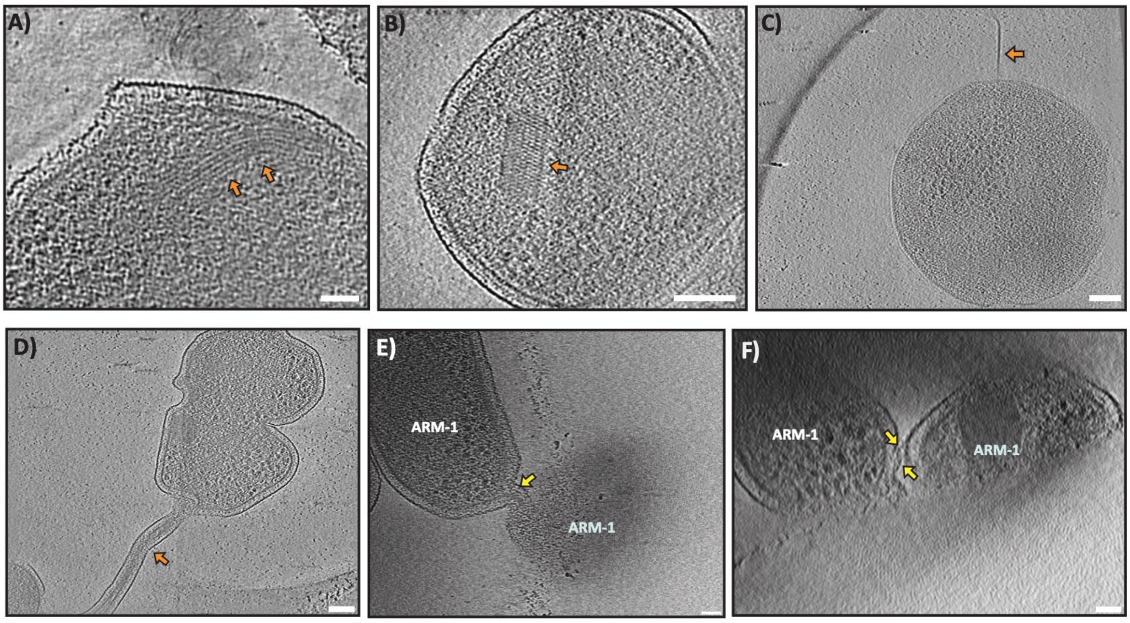

Supplementary Figure 10. Unknown features, morphologies, and interaction events observed in tomograms of ARM-1 cells. (A) Tomographic slice of an ARM-1 cell with orange arrows highlighting unknown filamentous structures in the cytoplasm, measuring 15-20 nm in diameter. (B) Tomographic slice of an ARM-1 cell with an orange arrow pointing to an unknown trapezoidal-shaped density observed in the cytoplasm. This density is seen in several ARM-1 tomographic reconstructions and, while its length and width appear irregular, it consistently presents a cross-hatched appearance. (C) Tomographic slice of ARM-1 with an orange arrow indicating a density corresponding to an archaellum. (D) Tomographic slice of an ARM-1 cell showing a tube-like extension of the S-layer and cytoplasm, as highlighted by the orange arrow. (E-F) Intercellular tubes connecting cytoplasms of two DPANN cells.

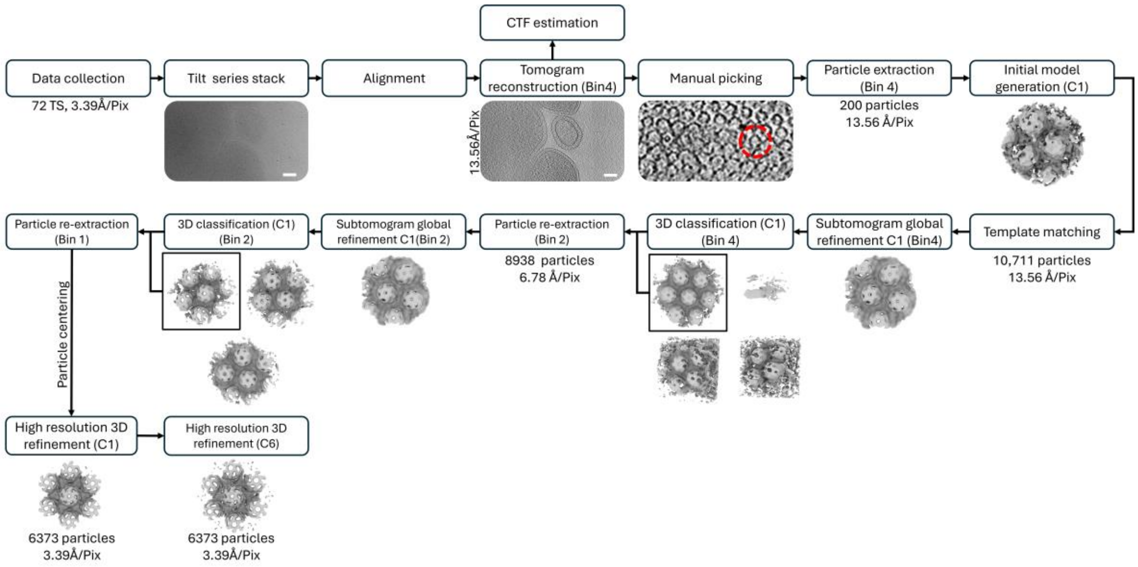

Supplementary Figure 11. Workflow for tomographic reconstruction and subtomogram averaging. Different steps during tomogram generation and subtomogram averaging are shown in this workflow.

References

1. Jumper, J. *et al.* Highly accurate protein structure prediction with AlphaFold. *Nature* **596**, 583–589 (2021).
2. Birmanns, S. & Wriggers, W. Multi-resolution anchor-point registration of biomolecular assemblies and their components. *Journal of Structural Biology* **157**, 271–280 (2007).
3. Pandurangan, A. P. & Topf, M. Finding rigid bodies in protein structures: Application to flexible fitting into cryoEM maps. *J Struct Biol* **177**, 520–531 (2012).
4. Pandurangan, A. P. & Topf, M. RIBFIND: a web server for identifying rigid bodies in protein structures and to aid flexible fitting into cryo EM maps. *Bioinformatics* **28**, 2391–2393 (2012).
5. Meng, E. C. *et al.* UCSF ChimeraX: Tools for structure building and analysis. *Protein Sci* **32**, e4792 (2023).
6. Quantitative analysis of cryo-EM density map segmentation by watershed and scale-space filtering, and fitting of structures by alignment to regions - PubMed. https://pubmed.ncbi.nlm.nih.gov/20338243/.

7. Birmanns, S., Rusu, M. & Wriggers, W. Using Sculptor and Situs for simultaneous assembly of atomic components into low-resolution shapes. *Journal of Structural Biology* **173**, 428–435 (2011).

8. Seemann, T. Prokka: rapid prokaryotic genome annotation. *Bioinformatics* **30**, 2068–2069 (2014).
