# Supplementary material for: Cell-to-cell interactions revealed by cryo-tomography of a DPANN co-culture system
Source: Nat Commun. 2024 Aug 16;15:7066. doi: 10.1038/s41467-024-51159-2 (PMC11329633; doi:10.1038/s41467-024-51159-2)
Supplement: Supplementary file 3 — Description of Additional Supplementary Files [file 41467_2024_51159_MOESM3_ESM.pdf]

Description of Additional Supplementary Files

File Name: Supplementary Movie 1

Description: Three-dimensional (segmented) view of an ARM-1 and AS-7 interaction showing intercellular proteinaceous tubes traversing between the host and ARM-1.

File Name: Supplementary Movie 2

Description: Three-dimensional (segmented) view of an ARM-1 and AS-7 interaction showing intercellular proteinaceous tubes traversing between the host and ARM-1 and unknown structural features inside ARM-1 cells.

File Name: Supplementary Movie 3

Description: Pseudo-atomic model of the AS-7 S-layer, showing the fit of the SlaA and SlaB AlphaFold models to the in situ AS-7 S-layer structure.

File Name: Supplementary Movie 4

Description: Structure of the contracted tube after segmentation, showing the cross-section of the tube from the perspective of inside the AS-7 cell and side on.

File Name: Supplementary Movie 5

Description: Tomogram movie through the z axis of an AS-7 cell, showing the presence of tube structures in the host cell in a pure host culture.

File Name: Supplementary Movie 6

Description: Tomogram movie moving through the z axis showing the membrane nanotube connections between ARM-1 cells.

Supplementary Data

File Name: Supplementary Data 1

Description: Proteomics analysis of AS-7 pure culture and AS-7 ARM-1 coculture using a two-tailed unpaired T-test.

File Name: Supplementary Data 2

Description: Presence loss analysis by comparative genomics analysis of Sulfolobaceae
