# Supplementary material for: Cell-to-cell interactions revealed by cryo-tomography of a DPANN co-culture system
Source: Nat Commun. 2024 Aug 16;15:7066. doi: 10.1038/s41467-024-51159-2 (PMC11329633; doi:10.1038/s41467-024-51159-2)
Supplement: Supplementary file 12 — Reporting Summary [file 41467_2024_51159_MOESM12_ESM.pdf]

nature portfolio

Corresponding author(s): Debnath Ghosal

Last updated by author(s): 15/07/2024

Reporting Summary

Nature Portfolio wishes to improve the reproducibility of the work that we publish. This form provides structure for consistency and transparency in reporting. For further information on Nature Portfolio policies, see our Editorial Policies and the Editorial Policy Checklist.

Statistics

For all statistical analyses, confirm that the following items are present in the figure legend, table legend, main text, or Methods section.

n/a	Confirmed	
☐	☒	The exact sample size (*n*) for each experimental group/condition, given as a discrete number and unit of measurement
☐	☒	A statement on whether measurements were taken from distinct samples or whether the same sample was measured repeatedly
☐	☒	The statistical test(s) used AND whether they are one- or two-sided<br>*Only common tests should be described solely by name; describe more complex techniques in the Methods section.*
☒	☐	A description of all covariates tested
☒	☐	A description of any assumptions or corrections, such as tests of normality and adjustment for multiple comparisons
☒	☐	A full description of the statistical parameters including central tendency (e.g. means) or other basic estimates (e.g. regression coefficient) AND variation (e.g. standard deviation) or associated estimates of uncertainty (e.g. confidence intervals)
☒	☐	For null hypothesis testing, the test statistic (e.g. *F*, *t*, *r*) with confidence intervals, effect sizes, degrees of freedom and *P* value noted<br>*Give P values as exact values whenever suitable.*
☒	☐	For Bayesian analysis, information on the choice of priors and Markov chain Monte Carlo settings
☒	☐	For hierarchical and complex designs, identification of the appropriate level for tests and full reporting of outcomes
☒	☐	Estimates of effect sizes (e.g. Cohen's *d*, Pearson's *r*), indicating how they were calculated

Our web collection on statistics for biologists contains articles on many of the points above.

Software and code

Policy information about availability of computer code

Data collection	Tomography data collection done with FEI Tomography 5 software. Massspec data collection was done with Orbitrap 480™ spectrometer.
Data analysis	Identification and LFQ analysis were accomplished using MaxQuant (v2.0.2.0) followed by Perseus (version 1.6.0.7). Gene conservation analysis used NCBI tBLASTn, and EGGNog mapper (2.1.9). Tomograms were aligned by IMOD (etomo-tomo3D-PEET) or EMAN2. Sub tomogram averages were made by EMAN2. Segmentation used ariande.ai (https://ariadne.ai/), Knossos (http://knossos.app/) and Blender (https://www.blender.org/). Subtomogram average model fitting and refinement used AlphaFold v2.0, matchpt collage (Situs package https://situs.biomachina.org/), RIBFIND, Flex-EM and Segger tool in ChimeraX.

For manuscripts utilizing custom algorithms or software that are central to the research but not yet described in published literature, software must be made available to editors and reviewers. We strongly encourage code deposition in a community repository (e.g. GitHub). See the Nature Portfolio guidelines for submitting code & software for further information.

Data

Policy information about availability of data

All manuscripts must include a data availability statement. This statement should provide the following information, where applicable:

- Accession codes, unique identifiers, or web links for publicly available datasets
- A description of any restrictions on data availability
- For clinical datasets or third party data, please ensure that the statement adheres to our policy

Subtomogram averages of the AS-7 S-layer and primed nanotube are available under accession codes EMD-42578 [https://www.emdataresource.org/EMD-42578] (In situ structure of AS-7 S-layer) and EMD-42579 [https://www.emdataresource.org/EMD-42579] (In situ structure of AS-7 primed tubes). The mass spectrometry proteomics data have been deposited to the ProteomeXchange Consortium with the dataset identifier PXD045678 [https://www.ebi.ac.uk/pride/archive/projects/PXD045678] (Proteomics analysis of host AS-7 cells interacting with DPANN ARM-1).

Research involving human participants, their data, or biological material

Policy information about studies with human participants or human data. See also policy information about sex, gender (identity/presentation), and sexual orientation and race, ethnicity and racism.

Reporting on sex and gender	NA
Reporting on race, ethnicity, or other socially relevant groupings	NA
Population characteristics	NA
Recruitment	NA
Ethics oversight	NA

Note that full information on the approval of the study protocol must also be provided in the manuscript.

Field-specific reporting

Please select the one below that is the best fit for your research. If you are not sure, read the appropriate sections before making your selection.

☒ Life sciences ☐ Behavioural & social sciences ☐ Ecological, evolutionary & environmental sciences

For a reference copy of the document with all sections, see nature.com/documents/nr-reporting-summary-flat.pdf

Life sciences study design

All studies must disclose on these points even when the disclosure is negative.

Sample size	NA
Data exclusions	NA
Replication	NA
Randomization	NA
Blinding	NA

Reporting for specific materials, systems and methods

We require information from authors about some types of materials, experimental systems and methods used in many studies. Here, indicate whether each material, system or method listed is relevant to your study. If you are not sure if a list item applies to your research, read the appropriate section before selecting a response.

Materials & experimental systems

n/a	Involved in the study
☒	☐ Antibodies
☒	☐ Eukaryotic cell lines
☒	☐ Palaeontology and archaeology
☒	☐ Animals and other organisms
☒	☐ Clinical data
☒	☐ Dual use research of concern
☒	☐ Plants

Methods

n/a	Involved in the study
☒	☐ ChIP-seq
☒	☐ Flow cytometry
☒	☐ MRI-based neuroimaging

Plants

Seed stocks	NA
Novel plant genotypes	NA
Authentication	NA
